# The MYST histone acetyltransferases are essential for gametophyte development in Arabidopsis

**DOI:** 10.1186/1471-2229-8-121

**Published:** 2008-11-28

**Authors:** David Latrasse, Moussa Benhamed, Yves Henry, Séverine Domenichini, Wanhui Kim, Dao-Xiu Zhou, Marianne Delarue

**Affiliations:** 1 Institut de Biotechnologie des Plantes, CNRS UMR8618, Université Paris-sud XI, 91405 Orsay, France

## Abstract

**Background:**

Histone acetyltransferases (HATs) play critical roles in the regulation of chromatin structure and gene expression. Arabidopsis genome contains 12 HAT genes, but the biological functions of many of them are still unknown. In this work, we studied the evolutionary relationship and cellular functions of the two Arabidopsis HAT genes homologous to the MYST family members.

**Results:**

An extensive phylogenetic analysis of 105 MYST proteins revealed that they can be divided into 5 classes, each of which contains a specific combination of protein modules. The two Arabidopsis MYST proteins, HAM1 and HAM2, belong to a "green clade", clearly separated from other families of HATs. Using a reverse genetic approach, we show that *HAM1 *and *HAM2 *are a functionally redundant pair of genes, as single Arabidopsis *ham1 *and *ham2 *mutants displayed a wild-type phenotype, while no double mutant seedling could be recovered. Genetic analysis and cytological study revealed that *ham1ham2 *double mutation induced severe defects in the formation of male and female gametophyte, resulting in an arrest of mitotic cell cycle at early stages of gametogenesis. RT-PCR experiments and the analysis of transgenic plants expressing the *GUS *reporter gene under the *HAM1 *or the *HAM2 *promoter showed that both genes displayed an overlapping expression pattern, mainly in growing organs such as shoots and flower buds.

**Conclusion:**

The work presented here reveals novel properties for MYST HATs in Arabidopsis. In addition to providing an evolutionary relationship of this large protein family, we show the evidence of a link between MYST and gamete formation as previously suggested in mammalian cells. A possible function of the Arabidopsis MYST protein-mediated histone acetylation during cell division is suggested.

## Background

Eukaryotic genomic DNA is packaged around octamers of histones to form the basic structural units of chromatin, the nucleosomes. Chromatin is the functional template for a variety of key biological processes, such as DNA replication, repair of DNA damage, recombination, and transcription.

Covalent modifications of the amino-terminal tails of the core histones affect nucleosome positioning and compaction, and therefore play pivotal roles in chromatin remodelling and in gene regulation. Histone modifications include acetylation, methylation, phosphorylation, ubiquitination, sumoylation and poly-ADP-ribosylation [1,2]. Among these modifications, acetylation of histones appears as a key switch for inter-conversion between permissive and repressive states of chromatin domains and as in other eukaryotes, histone acetylation and deacetylation play important roles in the regulation of plant gene expression. In general, hyperacetylation of histones relaxes chromatin structure and is associated with transcriptional activation, whereas hypoacetylation of histones induces chromatin compaction and gene repression, although a more sophisticated and nuanced chromatin language is likely to yield dynamic functional outcomes [3]. Acetylation of histones provides also an epigenetic marker for gene expression because it blocks association of heterochromatin-stabilising complexes [4].

The homeostatic balance of nucleosomal histone acetylation is maintained by antagonistic action of histone acetyltransferases (HAT) and histone deacetylases (HDAC), which are the best-characterized enzymes among histone modifier factors. In Arabidopsis, the HAT group contains 12 members divided into four classes based on sequence homology and mode of action: GNAT (Gcn5-related *N-*acetyltransferase), p300/CBP, TAF_II_250 and MYST (MOZ, YbF2, Sas2, Tip60-like) families [5,6].

Numerous reports have linked specific histone acetyltransferases to transcriptional regulation in Arabidopsis. For instance, we and other have shown that GCN5, plays a role in the regulation of numerous processes, including cold tolerance, floral development, embryonic cell-fate patterning, and light responsiveness [7-11]. HAF2, one of the two TAF_II_250 homologs in Arabidopsis is necessary for upregulating the transcription of light-induced genes [10,12]. HAC1, HAC5 and HAC12 of the CBP family have been shown to be involved in regulating flowering time [13]. Functions for other predicted Arabidopsis HATs have not yet been determined.

In mammals, the MYST family is the largest and most divergent. It has been intensely studied because of its broad conservation and biological significance. Experiments performed in the last few years show that MYST family proteins are involved in a wide range of cell function ranging from transcription activation and silencing, apoptosis, cell cycle progression, DNA replication or DNA repair with often a link to pathological disorder such as cancer (reviewed by [14-16]). For instance, Histone acetyltransferase bound to ORC (Hbo1) has been shown to interact both with ORC7 and with MCM2, essential proteins of the pre-replication complex (Pre-RC) [17,18] and to positively regulate pre-RC assembly and initiation of DNA-replication [19-21]. Another mammalian MYST protein, HIV tat-interacting protein 60 (Tip60), seems to be a functional homologue of the yeast protein Esa1 [22], which is the only essential HAT for yeast viability, playing a role in cell cycle progression [23]. It appears that this function has been conserved during evolution and several recent results provide evidence that Tip60 is a key protein in regulating cell cycle progression in higher organisms. Likewise, a broad range of functions has been ascribed to Tip60 as its involvement in DNA repair or regulation of apoptosis (reviewed by [15]). MORF (monocytic leukaemia zinc finger protein-related factor) may be involved in early mammalian gametogenesis [24], whereas the mouse orthologue, Querkopf, has been implicated in neural development and maintenance of neural stem cells [25]. At last, it has been recently shown, that Mof (Males absent on the first) is essential for progression of embryonic development in mice [26,27].

Two MYST family members (*HAM1 *and *HAM2*) are present in the *A. thaliana *genome [6]. They were demonstrated to possess an *in vitro *HAT activity specific for lysine 5 of histone H4 (H4K5) [28]. However, the relevance of such observations to the biological roles of *HAM1 *and *HAM2 *has not been addressed.

Here, we report on the phylogenetic analysis of MYST family, which appears clearly separate from other families of HAT. By analyzing loss-of-function lines in Arabidopsis, we show that the two members of this family are functionally redundant, and provide evidence that they are required post-meiotically for important cellular process during the formation of both the male and female gametes.

## Results

### Phylogenetic analysis of MYST proteins

Initially, members of the MYST group were classified as putative acetyltransferases based on a region in the MYST domain that is homologous to the canonical acetyl-CoA binding domain (motif A) found in GNAT superfamily acetyltransferases [29]. The *Arabidopsis *genome encodes two closely related MYST family proteins HAM1 and HAM2 (87.9% identity, 92.5% similarity in amino-acid sequences), also known as respectively HAG4 and HAG5 [6]. Wolfe data  postulated that *HAM1 *(*At5g64610*) and *HAM2 *(*At5g09740*) resulted from a duplication event, the α event according to [30], produced by a polyploidization in the Brassicaceae ancestor. The *Arabidopsis thaliana HAM1 *and *HAM2 *genes show a Ks value of 0.84. In their measure of divergence between duplicated genes, De Bodt et al. (2005) [31] conclude that the most recent polyploidization event corresponds to a modal Ks value between 0,7 and 0,9. This reinforce the previous observation that the *HAM1 *and *HAM2 *genes from *Arabidopsis *belong to duplicated segments produced by the most recent polyploidization event in the Brassicaceae ancestor.

**Figure 1:**
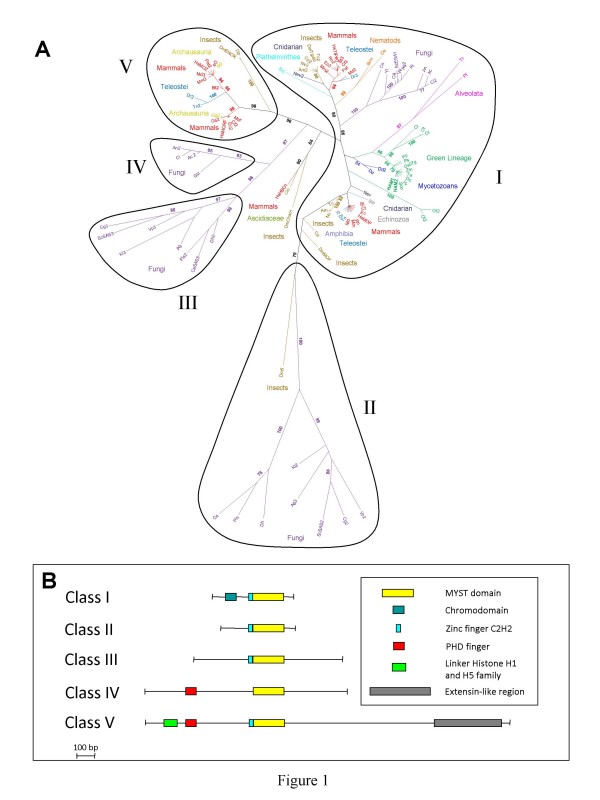
**Phylogenetic tree and domain organization of the MYST family of HAT**. (A) Numbers refer to the bootstrap values. The figure shows an unrooted phylogenetic tree based on an extensive search in available databases. The tree contains 105 sequences selected on a total of 130 obtaining by an extensive BLAST search. The plant lineage is shown in green, mammals are in red and fungi in purple. The MYST family is divided into five unrelated classes displaying similar domains organization. (B) Domain organization of the MYST proteins in the five classes previously defined in (A).

In order to identify the closest sequences to HAM1 and HAM2, a phylogenetic analysis of the MYST proteins was performed by an extensive search in available databases. Amino acid alignements of the MYST domain were used as the basis for classifying MYST proteins. Fig. 1A shows an unrooted phylogenetic tree illustrating the relationship between 105 MYST proteins (listed in Additional file 1) selected on a total of 130.

The MYST family is divided into five unrelated classes, i.e. not related by significant bootstrap values. The class I comprises proteins from the green lineage including the Arabidopsis HAM1 and HAM2 sequences, two clades of sequences from Mammals, Teleostei, Insects and Cnidarian, and clades of sequences from Alveolata, Fungi, Nematods and Plathelminthes. The class II (bootstrap 79%) groups sequences of Fungi plus one sequence of insect. Classes III and IV (bootstrap 98% and 93%) are exclusively composed of sequences from Fungi. The class V (bootstrap 96%) enclosed sequences from Teleostei, Archausauria, Mammals and Insects. Three sequences (from Human, Insect and Ascidiaceae) form a small additionnal group (bootstrap 84%).

We also noticed that the MYST proteins from the green lineage (Eudicots and Monocots Angiosperms, Gymnosperms, Bryophytes, Chlorophyceae and Prasinophyceae) lie within a cluster supported by a significant bootstrap value (86%). This cluster is associated both with two sequences of Alveolata and 2 sequences of *Dictyostelium discoideum*, as is frequently observed for sequences from plants. A second cluster of MYST sequence from Prasinophyceae (*Ostreococus*) is observed. The green lineage phylogenetic tree appears robust with highly significant bootstrap values (Additional file 2). Members of the MYST family of acetyltransferases possess several protein domains. Structural analysis by using Pfam and SMART tools revealed that within each previously defined group, domain organizations of the MYST proteins are highly similar (Fig. 1B). Class I members possess a chromodomain (PF 00385) in the amino-terminus and a zinc finger (C2H2 type) contiguous to a MOZ-SAS acetyltransferase domain (PF 01853). The structure is reduced to the zinc finger and MOZ-SAS domains in classes II and III. Class IV possesses a PHD finger (plant homeodomain zinc finger, PF 00628) in the amino-terminus and a central MOZ-SAS domain. Finally, sequences from class V were much longer, with a linker histone H1 and H5 family domain (PF 00538), one or two PHD finger domain, both at the amino terminus, a C2H2 type zinc finger, contiguous to the MOZ-SAS domain and an extensin-like region (PF04554) at the carboxy terminus. The observed domain organization and protein sizes reinforced the idea of existence of several distinct MYST subfamilies.

### Loss-of-Function mutations at the *HAM1 *and *HAM2 *loci

**Figure 2:**
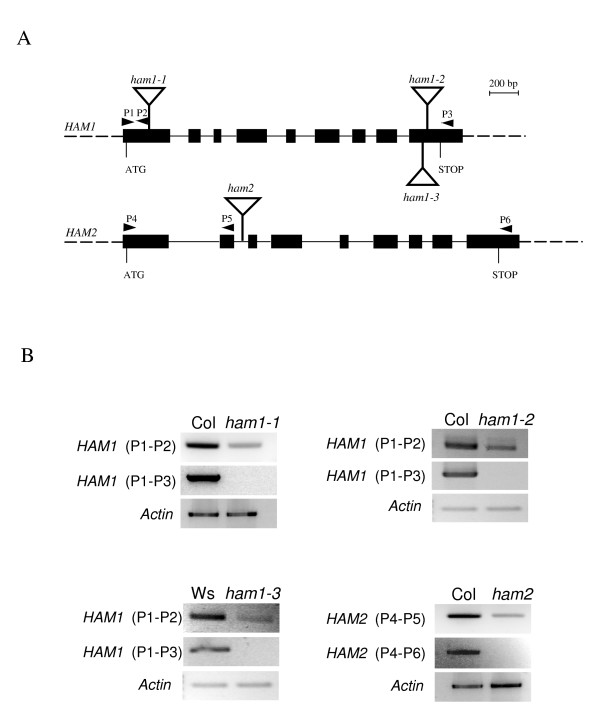
**Characterization of T-DNA insertion mutations within *HAM1 *and *HAM2***. (A) Schematic representation of the T-DNA insertions in the *HAM1 *and *HAM2 *genes. Black boxes and solid lines denote exons and introns, respectively. The filled triangles represent the six PCR primers used in RT-PCR (see "Methods"). (B) RT-PCR analysis of *HAM *genes expression in homozygous insertion mutants and wild-type seedlings with primer pairs P1–P2, P1–P3, P4–P5 and P4–P6 as indicated in (A).

In order to study the developmental function of plant *MYST *genes, a search in Arabidopsis T-DNA insertion mutant collections was performed. Three insertion lines in *HAM1 *gene were identified. *HAM1 *mutants, *ham1-1*, *ham1-2 *and *ham1-3*, disrupt the predicted coding region at 155, 1973 and 1959 bp in the genomic DNA and downstream of the initiation codon, respectively (Fig. 2A). A single insertion line, *ham2*, was identified in *HAM2 *gene. The T-DNA insert is located 809 bp downstream of the ATG (Fig. 2A). All these mutants are in Columbia-0 (Col-0) background, except for *ham1-3 *in the Wassilewskija (Ws) background.

Homozygous insertion plants were identified by PCR. RT-PCR experiments, with primers that span the insertion sites, were neither able to detect any *HAM1 *mRNA in the *ham1 *mutants nor *HAM2 *mRNA in *ham2 *mutant (Fig. 2B). There were no changes in the level of *HAM2 *mRNA in *ham1 *mutants lines compared with control. Similarly, *HAM1 *mRNA was unchanged in *ham2 *homozygous plants (Additional file 3). This indicates that the absence of one *HAM *transcript does not affect expression of the other.

We did not observed any abnormal phenotype in plants homozygous for either *ham1 *or *ham2 *mutations when grown under standard conditions. In addition, each of the mutant alleles displayed a normal Mendelian segregation ratio (data not shown). As *HAM1 *and *HAM2 *are closely related genes, functional redundancy might exist to prevent the appearance of a mutant phenotype in the homozygous mutant lines. Therefore, *ham1-1, ham1-2 *and *ham1-3 *homozygous plants were respectively crossed with *ham2 *homozygous plants to create double mutants. The resulting double-heterozygous F1 were allowed to be self-fertilized and individuals from the resulting progeny were genotyped using PCR. For each crosses, from more than 120 F2 individuals, no homozygous double mutants were detected. However, plants homozygous for insertion at one locus and heterozygous at the other were found. Such plants with either the genotype *HAM1/ham1*; *ham2/ham2 *or *ham1/ham1*; *HAM2/ham2 *were allowed to self-pollinate. For this F3 population, the expected frequency of *ham1ham2 *plants is 25%. From 166 seedlings from a *HAM1/ham1-1; ham2/ham2 *parent, no *ham1-1ham2 *plants were found (Additional file 4, line1). From 162 seedlings from a *ham1-1/ham1-1; HAM2/ham2 *parent, no *ham1-1ham2 *plants were found (Additional file 4, line 2). These data strongly indicate that *ham1ham2 *double mutant plants are not viable. Because F2 and F3 seeds appeared to be 100% viable, without seedling lethality after germination (data not shown), the loss of a double mutant plant must have occurred early during seed development or before fertilization and suggested that *HAM1 *and *HAM2 *have a redundant but important function for Arabidopsis embryo development and/or male/female gametophyte formation.

### *ham1ham2 *double mutant is affected in gametogenesis

If only the *ham1ham2 *developing seeds were not viable, then the progeny of self-fertilize *ham1/ham1*; *HAM2/ham2 *and *HAM1/ham1*; *ham2/ham2 *plants allowed to self-fertilize would segregate 2/1 for heterozygous and homozygous wild type at the heterozygous locus of the parent. However, the observation was that the percentage of heterozygous seedlings was much less than 67%, with only 32.5% heterozygous at the *HAM1 *locus and 18.5% heterozygous at the *HAM2 *locus (Additional file 4, lines 1 and 2). This deviation from a standard inheritance pattern implies death of more than just the double null developing seed.

**Figure 3:**
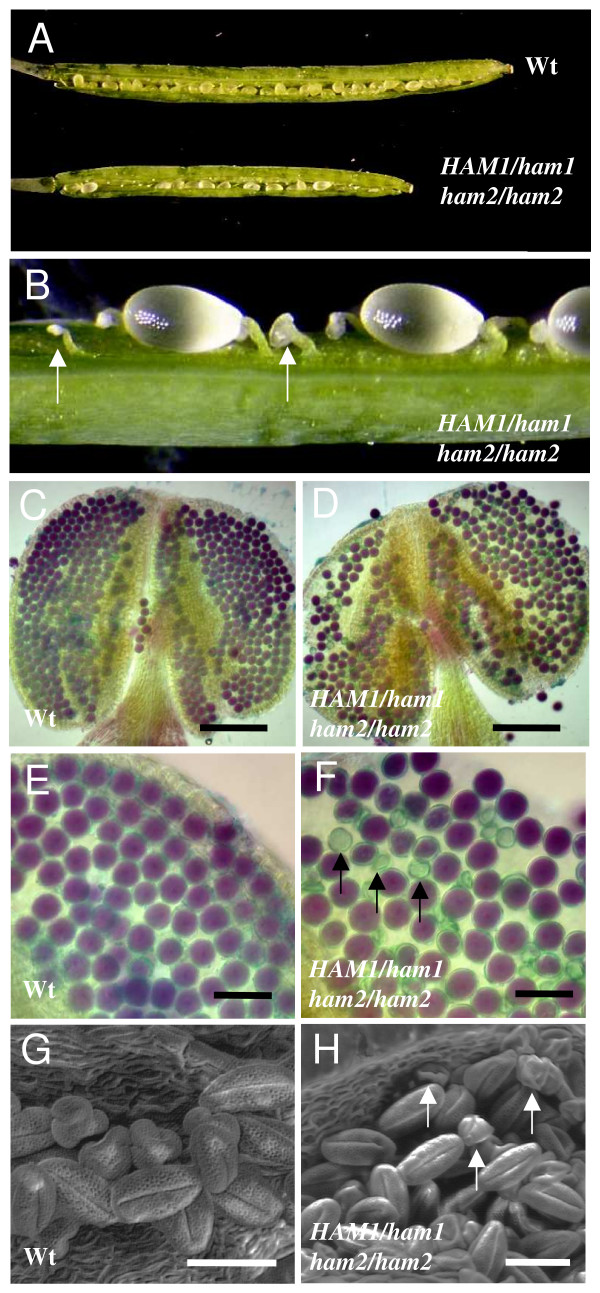
**Phenotypes induced by mutations in *HAM *genes**. (A) Comparison of a wild-type (Col-0, top) and *HAM1/ham1-1*; *ham2/ham2 *sesquimutant silique (bottom). (B) Detail of a sesquimutant silique showing the presence of aborted ovules (arrow). Viability of mature pollen grains in thesesquimutant (D) compared to wild-type (C). Pollen grains were isolated from the anthers of opened flowers and examined by Alexander staining. Viable pollen grains were stained as purple and dead pollen grains are dark green. Bars: 100 μm. High magnification detail of pollens from wild-type (E) and the *HAM1/ham1-1*; *ham2/ham2 *mutant (F) showing the presence of 50% wild type and 50% shrunken, aborted pollen (arrows) in the mutant anthers. Bars = 50 μm. Scanning electron microscopy of pollen grains collected from wild-type (G) and *HAM1/ham1-1*; *ham2/ham2 *anthers (H). Approximately one-half of pollen grains were abnormally developed in the sesquimutant anthers (arrows). Bars: 25 μm.

To confirm this hypothesis, the fertility of *ham1/ham1*; *HAM2/ham2 *and *HAM1/ham1*; *ham2/ham2 *mutants was determined after self-fertilization and compared to the wild-type. For different genotypes, the length of the siliques was reduced (Fig. 3A). For example, in the case of the *HAM1/ham1-1*; *ham2/ham2 *sesquimutant, the length of the siliques was 1.02 ± 0.10 cm compared to 1.44 ± 0.15 cm for the wild-type (n = 40). The number of seeds per silique was significantly reduced compared to wild-type siblings and was greater than the 25% loss expected by loss of the double mutant (Table 1). In addition, dissected siliques illustrated that unfertilized ovules were presents (Fig. 3B). This suggests that the loss of inheritance of the mutant allele occurred early, as a result of female gametophyte death.

**Table 1:** Genetic analysis and transmission of *ham1 *and *ham2 *mutations

	**Genotype of the parents** **Female × Male**	**Total numbers of developing seeds**	**Total numbers of unfertilized ovules**	**N**	**Percentages of unfertilized ovules**	***P*-value^a^**
1	*HAM1/HAM1;HAM2/HAM2 *selfed	280	11	291	3.8	0

2	*HAM1/HAM1;ham2/ham2 *selfed	294	10	304	3.3	0

3	*ham1-1/ham1-1;HAM2/HAM2 *selfed	318	7	325	2.2	0

4	*ham1-1/ham1-1;HAM2/ham2 *selfed	139	141	280	50.4	0.905

5	*HAM1/ham1-1;ham2/ham2 *selfed	161	157	318	49.4	0.822

6	*ham1-2/ham1-2;HAM2/HAM2 *selfed	362	12	374	3.2	0

7	*ham1-2/ham1-2; HAM2/ham2 selfed*	163	157	320	49.1	0.737

8	*HAM1/ham1-2;ham2/ham2 *selfed	196	201	397	50.6	0.801

9	*ham1-3/ham1-3; HAM2/HAM2 selfed*	302	12	314	3.8	0

10	*ham1-3/ham1-3;HAM2/ham2 *selfed	229	235	464	50.6	0.781

11	*HAM1/ham1-3;ham2/ham2 *selfed	112	115	227	50.7	0.841

12	WT × *ham1-1//ham1-1;HAM2/ham2*	125	4	129	3.1	0

13	*ham1-1/HAM1;ham2/ham2 *× WT	62	59	121	48.8	0.785

14	WT × *ham1-1/HAM1;ham2/ham2*	74	2	76	1.3	0

15	*ham1-1/ham1-1;HAM2/ham2 *× WT	52	55	107	51.4	0.771

Between 10 and 15 siliques from the main inflorescence stem of 5-week-old plants were manually dissected and scored for aborted ovules.

^a ^Two-tailed P-values represent the fit of the data to an expected segregation of 1:1 wild type: aborted.

To distinguish between arrested in embryo development and abnormalities in gametophytes, reciprocal crosses were performed to analyse inheritance via gametophytes. The null alleles, were successfully inherited from male gamete for both loci, although at a rate reduced from the expected 50% frequency (Additional file 4, lines 3 to 6). When pollen grains of the *HAM1/ham1-1*; *ham2/ham2 *or *ham1-1/ham1-1*; *HAM2/ham2 *mutants were used to pollinate the stigma of the wild-type female parent, the number of seeds was not significantly different from those observed in the wild-type (Table 1; lines 12 and 14). The frequency of inheritance of the *ham *allele from the male was 15.5% *for ham1 *and 9% for *ham2 *(Additional file 4, lines 4 and 6).

When *HAM1/ham1-1*; *ham2/ham2 *or *ham1-1/ham1-1*; *HAM2/ham2 *mutants were used as female parent, cleared siliques from these crosses showed that female gametophyte development was arrested in approximately one half of the ovules (Table 1; lines 13 and 15). This finding suggests that aborted ovules may correspond to a female gamete *ham1ham2*. Genotyping of the progenies confirmed that the inheritance of the *ham *alleles from the female was null for *ham1 *and *ham2 *(Additional file 4; lines 3 and 5).

To track the expression stage of the *ham1ham2 *mutations, we therefore examined the viability of pollens of the mutants. Pollens collected from wild-type and mutants bearing one copy of either *HAM1 *or *HAM2 *were stained with Alexander solution, which stained mature viable pollen grains as purple and dead or dying ones as dark green. The majority of examined pollens from wild-type were viable (Fig. 3C and 3E) with very few dead ones. In anthers of *ham1-1/ham1-1*; *HAM2/ham2 *mutants, however, only approximately 60% of pollens grains showed a staining pattern similar to that of the wild-type, the remaining were stained as dark green (n = 1400; Fig. 3D and 3F). These data are consistant with the reduce transmission of *ham2 *allele describe previously (Additional file 4). Morphologically, the dead pollens were misshapen and smaller (Fig. 3H), which could easily be distinguished from wild-type pollens (Fig. 3G).

**Figure 4:**
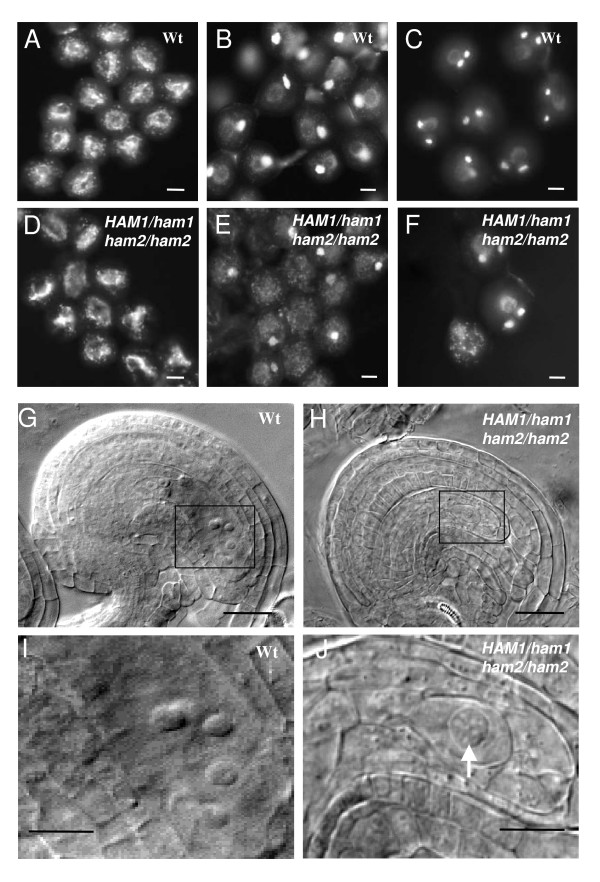
**Analysis of gametophyte development in *ham *sesquimutants**. (A-F) Analysis of pollen development by DAPI staining. Pollen grains were released from anthers, stained with DAPI, and visualized under a light microscope equipped with a UV fluorescent filter. At uni-nucleate stage, microspores of wild-type (A) and *HAM1/ham1-1*; *ham2/ham2 *plants (D) were indistinguishable. In contrast to wild-type microspores (B and C), *HAM1/ham1-1*; *ham2/ham2 *plants contain approximately one-half of arrested microspores (arrows) at the bicellular (E) and tricellular pollen stages (F). Bars: 10 μm. (G-J) Cleared whole mounts of wild-type and *ham *sesquimutant ovules. Mature ovules at stage G7 of wild-type (G) and *HAM1/ham1-1*; *ham2/ham2 *mutant (H). Bars: 40 μm. Images I and J are enlargements of boxed sections in G and H respectively. Bars: 20 μm. (I) Magnified section shows the presence of central cell nucleus, two synergid nuclei and the egg cell nucleus in wild-type ovule. (J) An ovule from *ham *sesquimutant at the same stage showing the presence of only one nucleus (white arrow). No other nuclei were visualized at other focal planes.

We further analyzed male gametophyte development in the double mutant by fluorescence and microscopy. The pollen grain is the male gametophyte in Angiosperms. During microsporogenesis, meiosis of the microspore mother cell produces a tetrad of microspores. After release from the tetrad, during microgametogenesis, each microspore goes through an asymmetric cell division, pollen mitosis I (PMI), to produce a bicellular pollen grain containing a generative cell and a much larger vegetative cell. Only the smaller generative cell undergoes a second round of cell division, pollen mitosis II, to produce two sperm cells [32,33]. Pollen grains collected from open flowers of both wild-type and mutants bearing one copy of either *HAM1 *or *HAM2*, were examined by staining with DNA-specific dye 4',6-diamidino-2-phenylindole (DAPI). When wild-type flowers were open, pollen grains were already mature, and they had two brightly stained sperm nuclei and a faintly stained vegetative nucleus (Fig. 4C). In single mutant plants, microsporogenesis proceeds normally (Additional file 5). By contrast, a part of pollens derived from *HAM1/ham1-1*; *ham2/ham2 *and *ham1-1/ham1-1*; *HAM2/ham2 *sesquimutant flowers displayed one large DNA mass (~36%, n = 280; Fig. 4E and 4F). These observations suggest that the degeneration of *ham1ham2 *double mutant pollens occurred mainly at the uninucleated stage before the first pollen mitosis (PM I).

On the female side, during megagametogenesis, meiosis of the megaspore mother cell gives rise to four megaspores, but three degenerates and one survives. This cell undergoes three round of mitosis to form a seven-celled mature embryo sac (female gametophyte) at female gametophyte stage 6 (FG6; [34]). The analysis of ovule development of *ham1 *and *ham2 *single mutants, using a chloral hydrate clearing protocol and Normarski optics, indicated that megasporogenesis occurred normally in single *ham *mutants (data not shown). In the ovules of sesquimutants bearing one copy of either *HAM1 *or *HAM2*, meiosis always resulted in a single surviving megaspore and as occurred in wild-type, only one of them survived. Initial abnormalities in megagametogenesis were observed only after the completion of meiosis. While half the ovules in both *HAM1/ham1-1; ham2/ham2 *and *ham1-1/ham1-1; HAM2/ham2 *siliques were mature showing a wild-type size and shape (Fig. 4G and 4I), the remaining ovules contained only one nucleus localized to the micropylar pole (corresponding to the stage FG1; Fig. 4H and 4J). They produced the 50% aborted ovules (Fig. 3B). These observations suggest that, as observed for the male gametophytic development, the degeneration of the ovule in *ham1ham2 *double mutants occurs after the uninucleated stage before the first mitosis.

### Expression pattern of *HAM *genes

**Figure 5:**
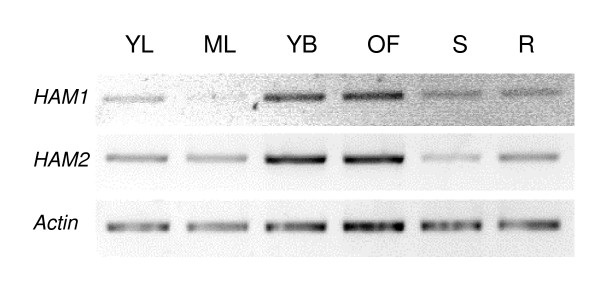
**RT-PCR detection of *HAM *mRNA in different organs**. *HAM1 *and *HAM2 *mRNA in wild-type (Col-0) were detected by RT-PCR with primer pairs P1-P3 (*HAM1*) and P4–P6 (*HAM2*). The different organs tested are young leaves (YL), mature leaves (ML), young floral buds (YB), open flowers (OF), stems (S) and roots (R). Actin mRNA levels detected by RT-PCR are shown as controls.

The *HAM1 *and *HAM2 *mRNA levels in different organs and tissues were too low to be detected by Northern blots. RT-PCR experiments were used to analyse the expression pattern of *HAM *genes. Fig. 5 shows that *HAM1 *and *HAM2 *genes displayed a similar expression pattern in the different tested organs, with higher expression in flowers compared to leaves, stems and roots and in younger growing leaves compared to mature leaves.

**Figure 6:**
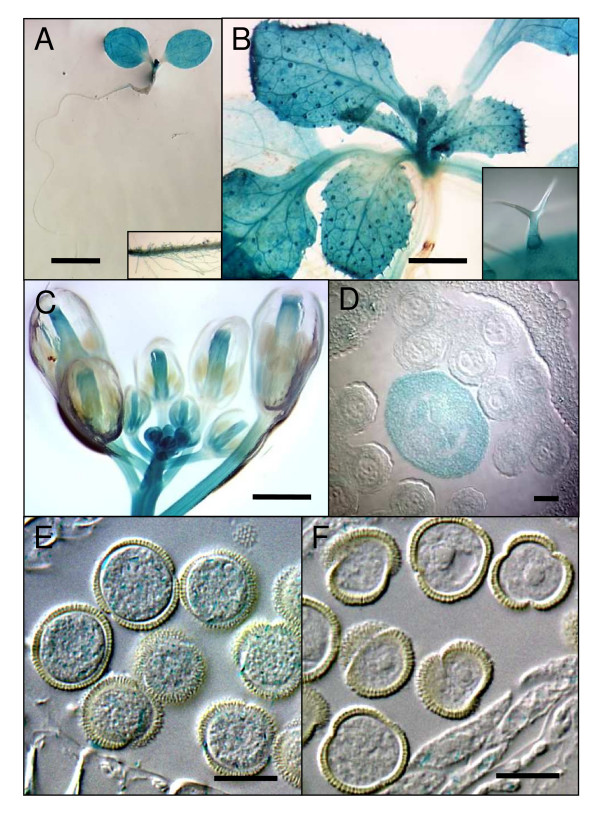
**Expression pattern of the *HAM::GUS *genes**. (A) Seedling of one-week-old harbouring a *HAM1::GUS *fusion grown *in vitro*. Boxed section represents a magnification of stained root hairs. Bar: 250 μm. (B) Three week-old plant grown *in vitro*. Boxed section represents a close-up view on a stained trichome. Bar: 1 mm. (C) Floral tissue of a 5-week-old plant. Bar: 1 mm. (D) Section of *HAM1::GUS *young floral bud at the stage of tetrad. Bar: 50 μm. (E-F) cleared whole mount of young (E) and mature pollen grains (F) showing the decreased of *GUS *expression during the pollen maturation. Bars: 25 μm.

Transgenic Arabidopsis lines were also generated to express the β-glucuronidase (*GUS*) reporter gene under the control of *HAM1 *or *HAM2 *promoter. About 1 kb DNA fragments encompassing the putative promoter regions of *HAM *genes were fused to the *GUS *coding sequence and these constructs were introduced into Col-0. A reproducible and overlapping expression pattern was found in three independent reporter lines for each promoter. Under standard growth conditions, promoter activity was detected in the shoot apex of the seedlings as well as in the cotyledons and leaves (Fig. 6A, B). In leaves, a patchy expression pattern was observed which corresponded to strong GUS staining at the basis of the trichomes (Fig. 6B). GUS activity was never detected in the hypocotyls and petiole. A faint GUS signal was occasionally detected in root hairs (Fig. 6A) but never in the internal root tissues. By contrast, GUS activity was strong in developing flowers, particularly in the anthers and gynoecia but not in mature flowers (Fig. 6C) in which a slight GUS activity was localized to the stigma. Transversal sections confirmed the GUS staining in developing gynoecia (Fig. 6D) and young pollens (Fig. 6E) and the absence of GUS expression in mature tricellular pollen grains (Fig. 6F).

## Discussion

The MYST family of HATs has been intensively studied because of its broad conservation and its involvement in multiple aspects of animal development (Reviewed in [15]). Although the HAT activity of the Arabidopsis MYST proteins HAM1 and HAM2 have been demonstrated *in vitro *[28], the *in vivo *roles of the proteins have not yet been addressed. Here, we initiated a functional characterization of the MYST gene family in Arabidopsis.

Growing genomic sequence data indicates that most eukaryotes had multiple MYST representatives. In order to gain insight into the origin and evolution of the MYST proteins, we combined phylogenetic sequence analysis and structural comparisons to determine the relationships among different members. Such phylogenetic analysis has been already performed by Sanjuan et al. [35], but it was realized with a limited number of data and do not provide precise links between clades. Our phylogenetic analysis suggests that Eukaryotes acquired 3 ancestral MYST sequences (Fig. 1A). It appears that one of these ancient sequences has been duplicated more recently, after the separation of Teleostei from Archausauria and Mammals. It seems to, that one of these sequences has been lost during early plant evolution, before the emergence of Prasinophyceae, followed by the lost of a second one before the emergence of Bryophytes. An alternative hypothesis would be a unique sequence at the beginning of the green lineage, with the acquisition of second sequence by green algae, followed by the lost of one of these sequences (type Ot2/Ol2) later during the green lineage evolution. This hypothesis suggests also the presence of three sequences before the emergence of Fungi and Metazoans.

The plant genomes were found to encode one or two MYST proteins whereas fungal genomes were found to have one to three, and animal genomes one to five. Thus, the number of plant MYST family is within the range found in other eukaryotic organisms but at the lower end of this range. The degree of evolutionary change differs significantly among MYST gene families. At one extreme, the MYST family has been amplified in Mammals and Drosophila as compared to two members in Arabidopsis. Moreover domain and phylogenetic analyses of the MYST-type proteins revealed only one class of these proteins in plants, as compared to 3 classes in Insects and Mammals. This suggests that plant may have conserved the functions of their ancestral homologues and that Arabidopsis MYST proteins may be involved in a wide range of cell functions which are assumed by different proteins in mammals. An important point is that we do not detect sequences of other HATs from Angiosperms inserted in the phylogenetic tree built with sequences of the MYST family. This suggests a very old age of the MYST sequences, which were clearly separated from other families of HAT.

In order to study the biological function of the two plant MYST proteins, we performed a genetic analysis using Arabidopsis mutants. The study has shown that HAM1 and HAM2 are functionally redundant and the presence of at least one functional *HAM *gene is required for the plant, as single *ham1 *or *ham2 *mutant plants are viable and have a wild-type phenotype, while no double mutant seedling was recovered. *ham1ham2 *double mutation was not inherited because it caused mitotic defects in the mega and microgametophyte development as observed in individuals that contained a single wild-type copy of either *HAM1 *or *HAM2*. During ovule development in *HAM1/ham1; ham2/ham2 *or *ham1/ham1; HAM2/ham2*, abnormalities were detected after meiosis but before the first mitotic division in megagametogenesis. They produced 50% of aborted ovules *ham1ham2*. On the male side, pollen meiosis also appeared normal, while microsporogenesis was arrested also after microspores release but before the first pollen mitosis. In contrast to megagametogenesis, part of the *ham1ham2 *male gametes were able to produce normal pollen and were inherited.

Thus, in Arabidopsis, HAM proteins are redundantly required post-meiotically for important cellular processes during gametophytic development. However, the identification of *HAM1/ham1; ham2/ham2 *and *ham1/ham1; HAM2/ham2 *individuals, and the data collected from outcrossing eliminated the possibility of a complete male gametophytic lethal phenotype, although there was some implication of high gametophytic lethality from the sesquimutant plants. Given that in both mutants, the two T-DNA insertion sites are located at the beginning of the genes, before the HAT domain, it is unlikely that the truncated proteins HAM1 and HAM2 retain a partial HAT activity in the mutants. One possible explanation for the genetic complexity is the diffusion of trace amounts of functional protein from surrounding heterozygous tissues into the gametes [36,37]. It is also possible that meiotic cells contain a pool of proteins or mRNA that allows several rounds of nuclear division to occur before the effect of the mutation can be observed [38].

A possible involvement of the MYST protein in the gametogenesis has been recently suggested in mammals. The two human MYST proteins, Tip60 and Mof, related to HAM (Fig. 1), are both very highly expressed during sperm development compared to other organs [15]. Human MYST4, also named MOZ2 or MORF is also localized into specialized cells of the ovary and testis and may contribute to important and specific acetylation events during gametogenesis [24]. However, these reports are based only on pattern of genes expression and protein localization. The phenotypes of Arabidopsis knock-out mutants, described here, are the first demonstration of an essential role of MYST proteins in gametogenesis.

Using transgenic plants expressing the GUS reporter gene, a strong promoter activity of *HAM *genes was observed in young flowers, particularly during gynoecium and anther development, which is consistent with the requirement of HAM1 and HAM2 for gametogenesis. However, GUS expression and RT-PCR results have shown that *HAM *genes expression was not restricted to cells involved in gametogenesis. An expression pattern was also observed during vegetative development. Interestingly, a GUS activity was detected in trichoblasts (root hairs and trichomes). We can note that despite the differences in morphology and distribution, this kind of cells is specified by a similar set of genes [39]. GUS activity was also detected in shoot apex, cotyledons and leaves but neither in the primary root meristem nor the emerging lateral roots. These results indicate that *HAM1 *and *HAM2 *expression occurs mainly in some, but not all, proliferative tissues. The broad expression pattern of *HAM *genes suggests that they may be involved in several aspects of development, rather than gametophyte-specific genes.

Taking these GUS data together with the abnormal nuclear division in *ham1ham2 *gametes it is tempting to speculate a role for the MYST family in the control of key cellular process such as cell cycle control. This essentiality of the MYST pathway in plants is consistent with data related to the functions of MYST proteins in other organisms [16]. The closest homologues of Arabidopsis MYST proteins in *Homo sapiens *are Tip60 and HsMOF (Fig. 1). Several recent papers provide evidence that Tip60 is a key protein in regulating cell cycle progression in mammals [15]. Tip60 is also intimately involved in the cellular response to DNA damage. Additionally, in Drosophila the histone acetyltransferase activity of Tip60 is specifically required for the exchange of histones during double-strand break repair [40]. We do not know whether plant HAM proteins are directly involved in these processes. Although the ontogeny of gametophyte development has been defined in Arabidopsis, the molecular mechanisms regulating cell cycle progression are not well understood especially concerning how micro- and macrospores pass through each phase of the cell cycle, such as the G1/S transition during the unicellular stage. A phenotype with defects in the formation of male and female gametophyte due to interphase arrest of mitotic cell cycle at early stages (FG1 stage of megagametogenesis and PMI stage of microgametogenesis) will facilitate our understanding of the determining factors of gametophyte development. A highly similar phenotype has been recently described for the *rhf1arhf2a *double mutants which are defective in two RING-finger E3 ligases that mediate the degradation of the meiosis-accumulated ICK4/KRP6 that is essential to ensure cell cycle progression during gametogenesis [41]. Likewise, it has been recently demonstrated that HAM1 and HAM2 preferentially acetylate histone H4 lysine 5 (H4K5) in a similar fashion to the yeast homologue, Esa1 [28,42]. During the S-phase, newly synthesized histones H4 are deposited in a diacetylated isoform (at lysine 5 and 12) and it appears to be a highly conserved phenomenon in a wide range of organisms including plants [43,44]. These results are in agreement with a potential role of *HAM1 *and *HAM2 *during the S-phase. With abnormal nuclear divisions in *ham1ham2 *gametes, it is tempting to speculate a role for the Arabidopsis MYST family in controlling key cellular processes as DNA replication. This hypothesis remains to be tested during further analysis.

## Conclusion

The work presented here reveals novel properties for Arabidopsis MYST HAT. In addition to providing an extensive phylogenetic analysis of this large protein family, we provide evidence of a link between MYST and gamete formation in both male and female organs. The getting of conditional mutants with an inducible mis-expression system and the identification of the partners of these factors would also provide new tools to study the implication of MYST at the level of cellular process.

## Methods

### Identification of T-DNA insertion mutants

*ham1-1 *and *ham2 *mutants carrying T-DNA insertions respectively within *HAM1 *(*At5g64610*; SALK_027726) and *HAM2 *(*At5g09740*; SALK_106046) were obtained from The Nottingham Arabidopsis Stock Center (NASC). *ham1-2 *was a GABI-KAT line (050B11) [45]. acquired from the NASC. *ham1-3 *(EHQ293) was obtained from the Versailles collection. The following PCR primers were used to genotype plants carrying these T-DNA insertions. *ham1-1 *F: 5'-ATGGTGTGCGAATCTATGACC-3'; *ham1-1 *R: 5'-TCAAGGTCAAGCTGTTCAAGC-3'; *ham1-2 *F: 5'-TTACAGGTGGGCAAGAAG-3'; *ham1-2 *R: 5'-ACCATCCAGACAAAAGATTCC-3'; *ham1-3 *F: 5'-AAGGAAGGGCTATGGCAAAT-3'; *ham1-3 *R: 5'-CGTTTTACCATCCAGACAAAAG-3' *ham2 *F: 5'-GTCGAAGAAGAGGAAAATGGG-3'; *ham2 *R: 5'-CATATGCCTTTGAAGCTGCTC-3'; T-DNA left border: 5'-CGATTTCGGAACCACCATCAAACAGGA-3'. All of the T-DNA mutants and wild-type plants in this study were from the Columbia ecotype Col-0 excepted for *ham1-3 *mutant in the Ws background.

### Growth Conditions

Arabidopsis plants were grown in a greenhouse under long-day conditions (16 h of light) at 19.5°C (day) and 17.5°C (night). For in vitro cultures, seeds were sown on 0.5 Murashige and Skoog medium, incubated at 4°C for 48 h, and then transferred to growth chambers.

### Genomic DNA and Total RNA extraction, PCR, RT-PCR

Arabidopsis leaves were used for genomic DNA extraction. PCR were carried out using the Promega GoTaq polymerase. Total RNA was isolated with TRIzol reagents (Invitrogen). First strand cDNA was synthesized from 3 μg of total RNA using ImProm-II reverse transcriptase (Promega). Polymerase chain reaction primers specific to the predicted cDNA sequences of each gene were used. For *HAM1*: P1, 5'-ATGGGATCGTCTGCGGATACA-3'; P2, 5'-GAATTCGTGAGAGCGAGTATCGCA-3'; P3, 5'-AGTCATCTAAGGATATGCAGA-3'. For *HAM2*: P4, 5'-CCTTTAACTCCTGATC-AAGCTAT-3'; P5, 5'-CTACAGCGCACTCTACTGAATC-3'; P6, 5'-GACAGCCCGCTTTACTTACACA-3'. For *Actin*: ACT-FP, 5'-ACCCAAAGGCCAACAGAGAGA-3'; ACT-RP, 5'-TGCTTGGTGCAAGTGCTGTGA-3'.

### Microscopy

To examine pollen viability, pollen grains were stained with Alexander solution [46]. The pollen nuclei were stained with DAPI according to a method already describe by [47]. For observations of ovules, siliques were fixed overnight using 3:1 acetic acid: ethanol and then washed with 70% ethanol before clearing in 8: 1: 2 chloral hydrate: glycerol: water. For scanning electron microscope (Hitachi S-3000) analysis, samples were slowly frozen at -18°C under a partial vacuum on the Peltier stage before observation under the environmental secondary electron detector mode.

### Promoters analysis

The *HAM1 *and *HAM2 *promoters sequences used contain 1 kb upstream of the ATG and were amplified from Arabidopsis genomic DNA with the 5'-GCAGAATTCTCATTGTAGGTAAAAGAA-3' and 5'-GGATCCTTCTTTAGTCGGGTCGGA-3' primers for *HAM1 *and the 5'-GCGAATTCGTCTAACAGACTAAACGT-3' and 5'-CGGATCCTTCTCGGTCGGGTCGGAG-3' primers for *HAM2*. The corresponding PCR fragments were cloned into PUC19 vector and then transferred upstream the *uidA *gene in the plant transformation vector pPR97. The constructs were used to transform Col plants by the floral dip method. Transgenic plants were obtained on kanamycin containing medium and later transferred to soil for optimal seed production. For analysis of GUS activity, samples were prefixed in 90% acetone at room temperature for 20 min, rinsed in staining buffer without 5-bromo-4-chloro-3-indolyl-_-D-glucoronic acid (X-Gluc), infiltrated with staining solution (100 mM sodium phosphate buffer, pH 7, 5 mM potassium ferrocyanide, 5 mM potassium-ferricyanide, 1 mM X-Gluc) under a vacuum for 15 min, and incubated at 37°C for 14 h. After a progressive dehydration in a series ethanol concentrations up to 70%, samples were cleared in 8: 1: 2 chloral hydrate: glycerol: water. For sections, the stained samples were fixed in FAA at 4°C overnight, and then embedded in Leica historesin. Semithin sections (3 μm) were cut and analyzed under a microscope.

### Data collection

We searched for MYST sequences from protein databases at NCBI. 130 protein sequences were downloaded from numerous genomes. This allows to analyse sequences from Plants, Algae, Mycetozoans, Insects, Teleostei, Mammals (including Monotremata and Marsupial), Amphibia, Cnidarian, Echinozoa, Fungi, Plathelminthes, Nematods, Alveolata, Archausauria and Ascidiaceae. Bacteria do not have counterpart to the MYST proteins.

### Alignment and phylogenetic analysis

The amino-acid alignment was conducted using Clustal [48,49] with default parameters. The generated alignment was adjusted manually. Amino acid alignments of the MYST domain were used as the basis for classifying MYST proteins. The unrooted tree was created using the PhYML algorithm and the maximum likelihood method [50]. To assess support for each node, bootstrap analysis were performed using 500 bootstrap replicates. A bootstrap value of 70% is likely to be correct at the 95% level, and bootstrap values higher than 70% were taken as sufficient evidence for grouping.

### Structural analyses

To search for domain organization in the MYST proteins, we analyzed the sequences in Pfam [51], Prosite [52] and SMART [52] databases.

### Divergence time between sequences

To assess the age of the divergence between sequences, we estimated the level of synonymous substitutions (Ks) between the *Arabidopsis thaliana HAM1 *and *HAM2 *genes. After removing gaps in the nucleotide alignment, per-site synonymous (Ks) and nonsynonymous (Ka) substitution rates were calculated using PAL2NAL [54].

## Authors' contributions

DL carried out mutant analysis and reporter studies, MB performed cloning for reporter studies, YH performed the phylogenetic analysis, SD participated in the cytological and microscopically studies, WK participated in the genotyping of T-DNA insertion lines, D-X Z participated in the design of study and helped to draft the manuscript, MD conceived the study, and participated in its design and coordination and wrote the manuscript. All authors read and approved the final manuscript.

## Supplementary Material

Additional file 1 **List of the 105 proteins belonging to the MYST family used in our phylogenetic analysis.** Click here for file

Additional file 2 **Phylogenetic tree of the plant and Mycetozoa MYST protein**. Click here for file

Additional file 3 **RT-PCR analysis of *HAM1 *gene expression in *ham2 *homozygous mutant and of the *HAM2 *gene expression in the *ham1 *homozygous mutant.** Click here for file

Additional file 4 **Segregation and inheritance of *ham *T-DNA alleles.** Numbers represent plants genotyped by PCR. *ham1 *represent the *ham1-1 *allele. Het represents plants that are heterozygous at the indicated locus (Freq. Obs., frequency observed). TE, transmission efficiency of the mutant allele = number of mutant alleles/number of total alleles. Click here for file

Additional file 5 **Meiotic spreads of wild-type (A to C) and *ham *sesquimutant (D to F).** Meiotic spreads of wild-type (A to C) and *ham *sesquimutant (D to F). No difference were detected in wild-type and mutant meiocytes during prophase I (pachytene: A and D), telophase I (B and E) and telophase II (C and F). Bars: 10 μm. Click here for file
