# Supplementary material for: The MYST histone acetyltransferases are essential for gametophyte development in Arabidopsis
Source: BMC Plant Biol. 2008 Nov 28;8:121. doi: 10.1186/1471-2229-8-121 (PMC2606689; doi:10.1186/1471-2229-8-121)
Supplement: Additional file 1 — List of the 105 proteins belonging to the MYST family used in our phylogenetic analysis. [file 1471-2229-8-121-S1.doc]

**Supplemental Data 1:**

Genes Organisms Clades Accession numbers

Ac *Ajellomyces capsulatus* Fungi XP_001543929.1

Ac2 *Ajellomyces capsulatus* Fungi XP_001544545.1

Ag *Ashbya gossyypii*  Fungi NP_983638

Ag2 *Ashbya gossyypii*   Fungi NP_983540.1

Ag3 *Ashbya gossyypii*  Fungi NP_985768.1

Am *Apis mellifera*  Insecta XP_625075.1

Am2 *Apis mellifera*  Insecta XP_396552.31

An *Aspergillus niger*  Fungi XP_001400518.1

An2 *Aspergillus niger* Fungi XP_001401940.1

HAM1 *Arabidopsis thaliana* Angiosperms NP_201266.1

HAM2 *Arabidopsis thaliana* Angiosperms NP_196536.1

Brm *Brugia malayi* Nematoda XP_001896351.1

Bt *Bos taurus* Mammals NP_001098953.1

Bt2 *Bos taurus* Mammals XP_874495

Bt3 *Bos taurus* Mammals XP_581934.2

Ca *Candida albicans* Fungi XP_719657

CaSAS3 *Candida albicans* Fungi EAK99276.1

Ce *Caenorhabditis elegans* Nematoda NP_504796.1

Cf *Canis familiaris* Mammals XP_536904.2

Cf2 *Canis familiaris*  Mammals XP_864262.1

Cf3 *Canis familiaris* Mammals XP_540849.2

Cg *Candida glabrata* Fungi XP_447865.1

Cg2 *Candida glabrata* Fungi XP_448389

Cg3 *Candida glabrata* Fungi XP_444951

Ci *Coccidioides immitis* Fungi XP_001247753.1

Ci2 *Coccidioides immitis* Fungi XP_001246984.1

Cio *Ciona intestinalis* Ascidiaceae NP_001071919.1

Cn *Cryptococcus neoformans* Fungi XP_568893.1

Cp *Culex pipiens* Insecta XP_001847149.1

Cp2 *Culex pipiens*  Insecta XP_001842875.1

Cr *Chlamydomonas reinhardtii* Chlorophyceae XP_001695757.1

Dc *Daucus carota* Angiosperms BAA32822.1

Dd *Dictyostelium discoideum* Mycetozoa XP001732993.1

Dd2 *Dictyostelium discoideum* Mycetozoa XP_643810.1

Dh *Debaryomyces hanseni* Fungi XP_461928.1

Dh2 *Debaryomyces hanseni* Fungi XP_456657

Dm5 *Drosophila melanogaster* Insecta CG1894

DmChameau *Drosophila melanogaster* Insecta CG5229

DmEnok *Drosophila melanogaster* Insecta NP_523838

DmMOF *Drosophila melanogaster* Insecta ABU97221.1

DmTIP60 *Drosophila melanogaster* Insecta CG6121

Dp *Drosophila pseudoobscura* Insecta XP_001360901

Dr *Danio rerio* Téléostei AAI55278.1

Dr2 *Danio rerio*  Téléostei CAM13411.1

Dr3 *Danio rerio* Téléostei NP_001013327

Ec *Equus caballus* Mammals XP_001500799.1

Ec2 *Equus caballus* Mammals XP_00150400.1

Ec3 *Equus caballus* Mammals XP_001494234

Gg *Gallus gallus*  Archosauria XP_424402.2

Gg2 *Gallus gallus*  Archosauria XP_421609.2

Giz *Gibberella zeae* Fungi XP_388657.1

HsHBO1 *Homo sapiens* Mammals NP_008998

HsMOF *Homo sapiens*  Mammals Q9H7Z6

HsMORF *Homo sapiens* Mammals AF119230.1

HsMOZ *Homo sapiens* Mammals NP_001092882.1

HsTIP60 *Homo sapiens* Mammals NP_874369.1

Kl *Kluweromyces lactis* Fungi XP-455590.1

Kl2 *Kluweromyces lactis* Fungi XP_455864.1

Kl3 *Kluweromyces lactis* Fungi XP_454852

Maf *Macaca fascicularis* Mammals BAB72094.1

Md *Monodelphis domestica*  Mammalia XP_0013722001

Md2 *Monodelphis domestica* Mammalia P_001365749.1

Md3 *Monodelphis domestica* Mammalia XP_001373063.1

Mm *Mus musculus*  Mammals NP_080646.1

Mm2 *Mus musculus* Mammals XP_001113008

Mm3 *Mus musculus* Mammals NP_001074618.1

Nev *Nematostella vectensis* Cnidarian XP_001631947.1

Nev2 *Nematostella vectensis* Cnidarian XP_001627034

Nv *Nasonia vitripennis* Insecta NP_001600240.1

Nv2 *Nasonia vitripennis* Insecta XP_001606507.1

Oa *Ornitorhynchus anatinus* Mammalia XP_001509833.1

Oa2 *Ornitorhynchus anatinus*  Mammalia XP_001506182.1

Ol *Ostreococcus lucimarinus* Prasinophyceae XP_001419629.1

Ol2 *Ostreococcus lucimarinus* Prasinophyceae XP_001419529.11

Os07g *Oryza sativa*  Angiosperms NP_001060338.1

Ot *Ostreococcus tauri* Prasinophyceae CAL55531.1

Ot2 *Ostreococcus tauri* Prasinophyceae CAL55320.1

Pat *Pan troglodytes* Mammals XP_511869.2

Pat2 *Pan troglodytes* Mammals XP_51973.21

Pis *Pichia stipitis* Fungi XP_001382253.2

Pis2 *Pichia stipitis* Fungi XP_001386562

Pp *Physcomitrella patens* Bryophyta XP_001754279.1

Ps *Picea sitchi* Gymnosperms ABK24440.1

Pt *Paramecium tetraaurelia* Alveolata XP_001453783.1

Rn *Rattus norvegicus*  Mammals NP_001017378.1

Rn2 *Rattus norvegicus* Mammals EDL33154

ScESA1 *Saccharomyces cerevisiae*  Fungi NP_014887.1

ScSAS2 *Saccharomyces cerevisiae* Fungi CAA88552.1

ScSAS3 *Saccharomyces cerevisiae* Fungi CAA80794.1

Scj *Schistosoma japonicum* Platyhelminthes AAW26043.1

Soc *Solanum chacoense* Angiosperms AAQ24535.1

Stp *Strongylocentrotus purpuratus* Echinozoa XP_790129.2

Ta *Triticum aestivum*  Angiosperms ABG43094.1

Tc *Tribolium castaneum* Insecta XP_968431.1

Tc2 *Tribolium castaneum* Insecta XP_975410.1

Tn *Tetraodon nigroviridis* Teleostei CAG02259.1

Tn2 *Tetraodon nigroviridis* Teleostei CAG09717.1

Tt *Tetrahymena thermophila* Alveolata XP_001011213.1

Vp *Vanderwaltozyma polyspora* Fungi XP_001646190.1

Vp2 *Vanderwaltozyma polyspora* Fungi XP_001646720.1

Vp3 *Vanderwaltozyma polyspora* Fungi XP_001644288

Vv *Vitis vinifera*  Angiosperms CA061794.1

Xt *Xenopus tropicalis* Amphibia CAJ82114.1

Yl *Yarrowia lipolytica* Fungi XP_503552.1

Zm *Zea mays*  Angiosperms NP_001105074.1
