# Supplementary material for: The MYST histone acetyltransferases are essential for gametophyte development in Arabidopsis
Source: BMC Plant Biol. 2008 Nov 28;8:121. doi: 10.1186/1471-2229-8-121 (PMC2606689; doi:10.1186/1471-2229-8-121)
Supplement: Additional file 2 — Phylogenetic tree of the plant and Mycetozoa MYST protein. [file 1471-2229-8-121-S2.ppt]

## Slide 1
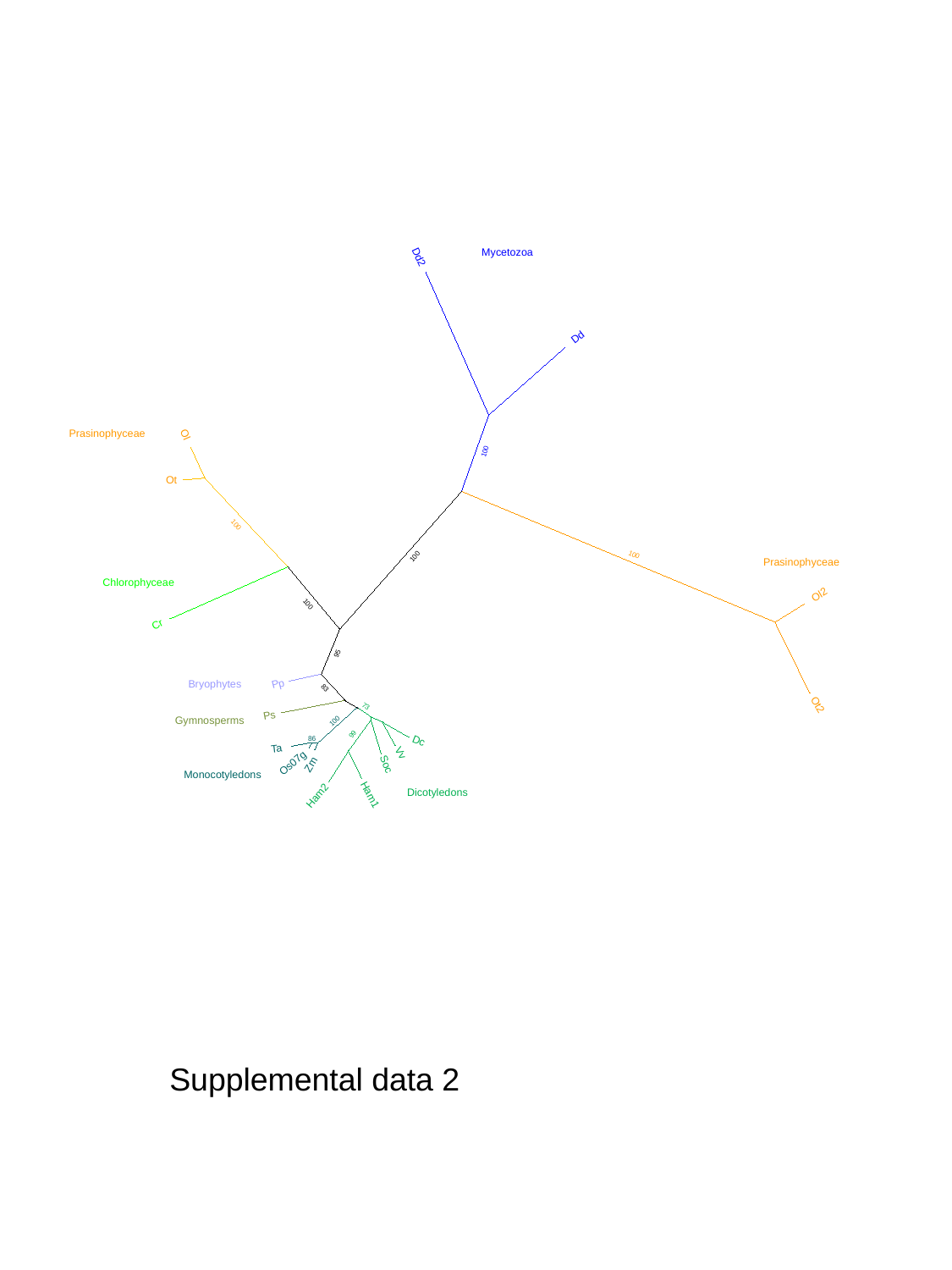

Mycetozoa
Dd2
Dd
Prasinophyceae
Ol
100
Ot
100
100
100
Prasinophyceae
Chlorophyceae
Ol2
100
Cr
95
Pp
Bryophytes
83
Ot2
73
Ps
Gymnosperms
100
99
86
Dc
Ta
Vv
Zm
Os07g
Soc
Monocotyledons
Dicotyledons
Ham1
Ham2
Supplemental data 2
