# Supplementary figures and images for: The MYST histone acetyltransferases are essential for gametophyte development in Arabidopsis
Source: BMC Plant Biol. 2008 Nov 28;8:121. doi: 10.1186/1471-2229-8-121 (PMC2606689; doi:10.1186/1471-2229-8-121)

## Slide 1
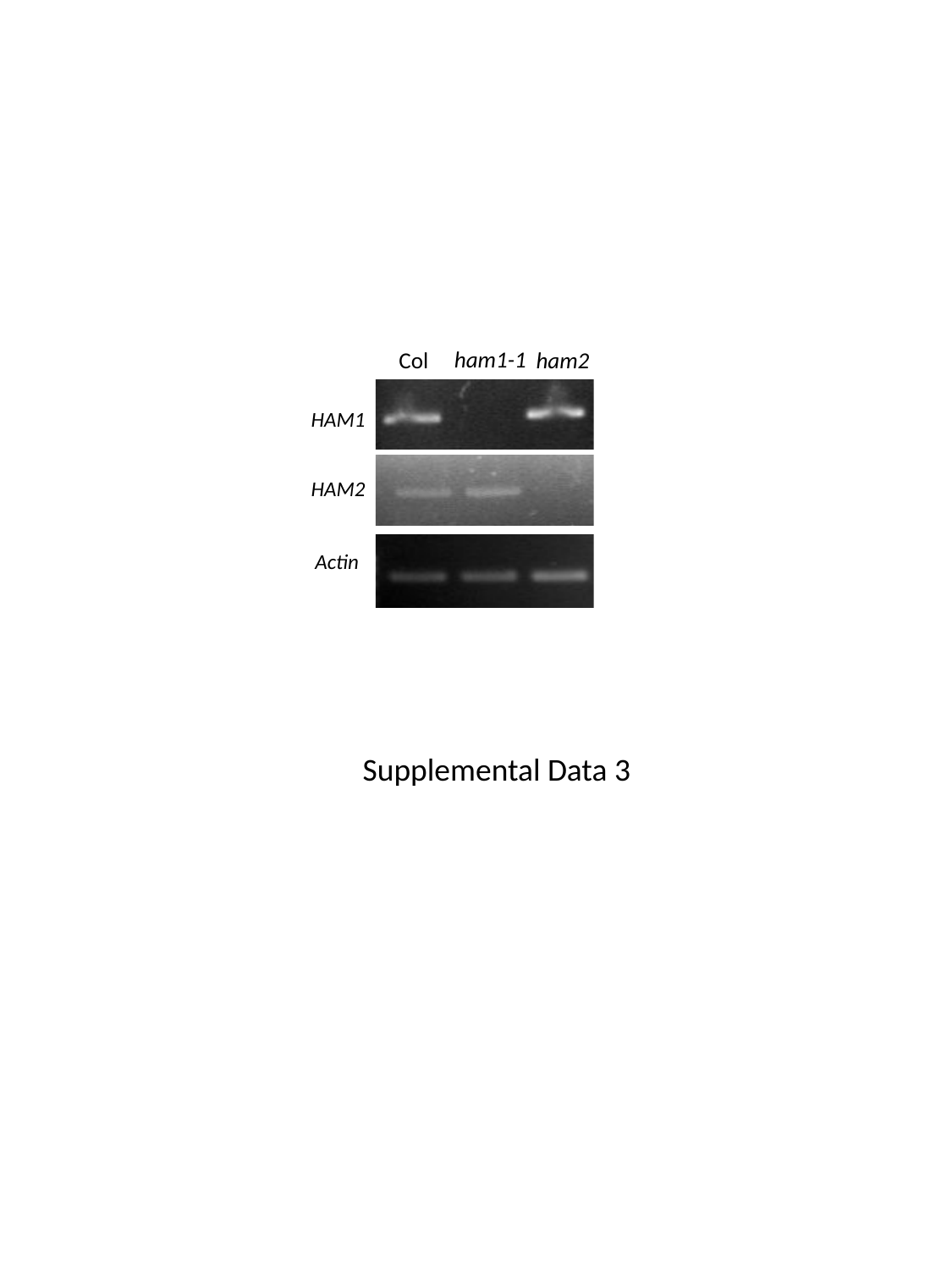

ham1-1
Col
ham2
HAM1
HAM2
Actin
Supplemental Data 3

Supplement: Additional file 3 — RT-PCR analysis of HAM1 gene expression in ham2 homozygous mutant and of the HAM2 gene expression in the ham1 homozygous mutant. [file 1471-2229-8-121-S3.ppt]

## Slide 1
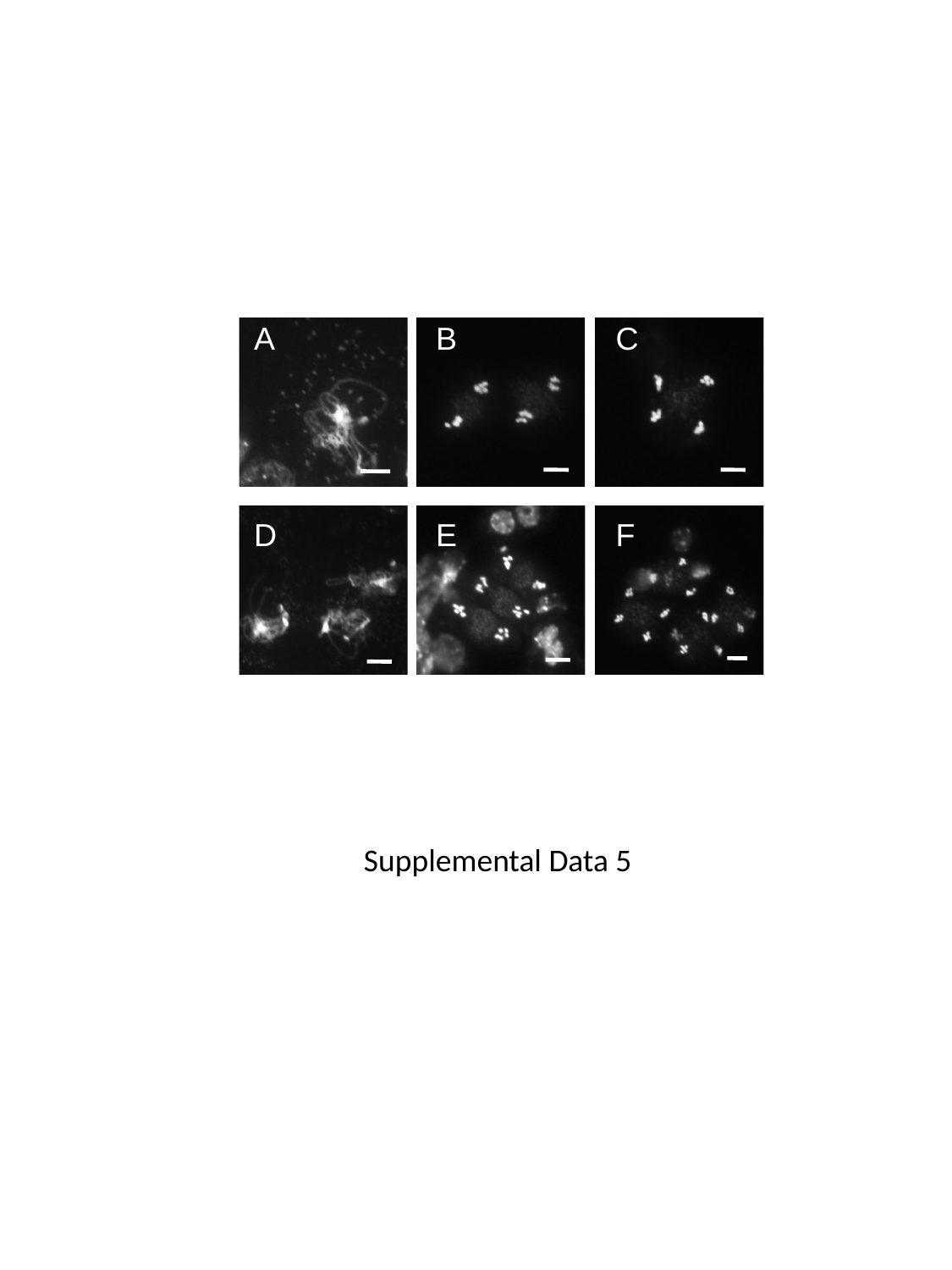

A
B
C
D
E
F
Supplemental Data 5

Supplement: Additional file 5 — Meiotic spreads of wild-type (A to C) and ham sesquimutant (D to F). Meiotic spreads of wild-type (A to C) and ham sesquimutant (D to F). No difference were detected in wild-type and mutant meiocytes during prophase I (pachytene: A and D), telophase I (B and E) and telophase II (C and F). Bars: 10 μm. [file 1471-2229-8-121-S5.ppt]
