# Supplementary material for: The MYST histone acetyltransferases are essential for gametophyte development in Arabidopsis
Source: BMC Plant Biol. 2008 Nov 28;8:121. doi: 10.1186/1471-2229-8-121 (PMC2606689; doi:10.1186/1471-2229-8-121)
Supplement: Additional file 4 — Segregation and inheritance of ham T-DNA alleles. Numbers represent plants genotyped by PCR. ham1 represent the ham1-1 allele. Het represents plants that are heterozygous at the indicated locus (Freq. Obs., frequency observed). TE, transmission efficiency of the mutant allele = number of mutant alleles/number of total alleles. [file 1471-2229-8-121-S4.doc]

**Supplemental data 4 :**

|  |  |  | *ham2/ham2* | | | *ham1/ham1* | | | *HAM2/ham2* | | *HAM1/ham1* | | | Het Freq. Obs.  (%) | TE |
| --- | --- | --- | --- | --- | --- | --- | --- | --- | --- | --- | --- | --- | --- | --- | --- |
|  | Female Parent | Male Parent | *HAM1/ham1* | *HAM1HAM1* | *ham1/ham1* | *HAM2/ham2* | *HAM2/HAM2* | *ham2/ham2* | *HAM1/ham1* | *HAM1/HAM1* | *HAM2/ham2* | *HAM2/HAM2* | n |
| 1 | *HAM1/ham1*;  *ham2/ham2* | *HAM1/ham1*;  *ham2/ham2* | 54 | 112 | 0 |  |  |  |  |  |  |  | 166 | 32.5 | 16.2 |
| 2 | *ham1/ham1*;  *HAM2/ham2* | *ham1/ham1*;  *HAM2/ham2* |  |  |  | 30 | 132 | 0 |  |  |  |  | 162 | 18.5 | 9.25 |
| 3 | *HAM1/ham1*;  *ham2/ham2* | WT |  |  |  |  |  |  | 0 | 124 |  |  | 124 | 0 | 0 |
| 4 | WT | *HAM1/ham1*;  *ham2/ham2* |  |  |  |  |  |  | 32 | 72 |  |  | 104  0 | 31  0 | 15.5 |
| 5 | *ham1/ham1*;  *HAM2/ham2* | WT |  |  |  |  |  |  |  |  | 0 | 113 | 113 | 0 | 0 |
| 6 | WT | *ham1/ham1*;  *HAM2/ham2* |  |  |  |  |  |  |  |  | 27 | 122 | 149 | 18.1 | 9.05 |
